# The Health Heterogeneity of and Health Care Utilization by the Elderly in Taiwan

**DOI:** 10.3390/ijerph110201384

**Published:** 2014-01-27

**Authors:** Li-Fan Liu

**Affiliations:** Institute of Gerontology, College of Medicine, National Cheng Kung University, Tainan 704, Taiwan; E-mail: lilian@mail.ncku.edu.tw; Tel./Fax: +886-63-028-173

**Keywords:** aged, delivery of health care, frail elderly, comorbidity, ethnicity

## Abstract

A good understanding of the health heterogeneity of elderly people, their characteristics, patterns of health care utilization and subsequent expenditures is necessary to adequately evaluate the policy options and interventions aimed at improving quality and efficiency of care for older people. This article reviews studies that used Latent Class Analysis to identify four health profiles among elderly people in Taiwan: High Comorbidity (HC), Functional Impairment (FI), Frail (FR), and Relatively Healthy (RH). Variables associated with increased likelihood of being in the FR group were older age, female gender, and living with one’s family, and these also correlated with ethnicity and level of education. The HC group tended to use more ambulatory care services compared with those in the RH group. The HC group tended to be younger, better educated, and was more likely to live in urban areas than were people in the FI group. The FI group, apart from age and gender, was less likely be of Hakka ethnicity and more likely to live with others than were individuals in the RH group. The FI group had relatively high probabilities of needing assistance, and the FR group had higher healthcare expenditures. A person-centered approach would better satisfy current healthcare needs of elderly people and help forecast future expenditures.

## 1. Introduction

Taiwan’s population is aging faster than that in most other developed countries [1] and now faces an increased prevalence of age-related diseases and escalating healthcare costs. At the end of 2012, 11.15% of the population was 65 years old or older, and it is estimated that the elderly population will double to 20% during the next two decades [1]. In Taiwan, life expectancy among elderly people is currently 76.4 years among males and 82.8 years among females [1]. Due to the multiple comorbid chronic conditions and complex care needs of older people, it is highly likely that older Taiwanese will seek health care related services, including frequent visits to multiple physicians and will also try to meet their long-term health care needs in the acute care system, which is supported by the National Health Insurance (NHI) program. For elderly people, multiple diseases and comorbidities have become the norm, and the number of people living for decades with chronic diseases is increasing worldwide [2]. 

To deal with the healthcare needs of elderly people and their subsequent use of healthcare services, it is increasingly important to shift our traditional view of diseases and syndromes to a person-centered approach, which takes multiple observed health indicators into account to describe health heterogeneity more comprehensively. Approaching healthcare needs by addressing elements of the mind, body, and spirit, as exemplified by integrative medicine in America, will better reflect the healthcare needs of elderly patients, and has emerged as a potential solution to America’s current healthcare crisis [3]. The overall aging of society has also placed a greater emphasis on the importance of holistic health, as specified by the World Health Organization (WHO) [4]. Thus, a holistic health framework is needed to capture disparate diseases and health conditions and their intricate relationships [5]. A better understanding of the health heterogeneity of elderly people, their patterns of health care use, and subsequent expenditures is necessary to adequately evaluate the policy options and interventions aimed at improving quality and efficiency of care for older people.

To identify the health profiles of elderly people in Taiwan, a model based on Latent Class Analysis (LCA) was used. This model utilizes the methodology that is part of the Mplus program [6], and is suitable for dealing with the issue of heterogeneity. LCA provides an empirical method that examines the interrelationships among health indicators and characterizes the underlying set of mutually exclusive latent classes that account for the observed indicators [7]. Based on maximum-likelihood, LCA estimates conditional item response probabilities and health latent class probabilities. Individuals are then assigned to a single class based on their highest LCA [6]. Model fit of LCA was assessed with Bayesian information criteria (BIC) and Lo-Mendell-Rubin likelihood ratio tests (LMR-LRT). LMR-LRT compares improvement in fit (*p* < 0.001) between sequential class models through approximation of the LRT distribution. An entropy measure was used to assess how well the model predicted class membership, given the observed health indicators. Entropy values range from 0 to 1, and higher values are preferred. Due to its person-centered approach, LCA can be used to reveal health profiles of even the smallest unobserved groups of individuals (*i.e.*, latent classes), and this can adequately account for the associations among observed health dimensions. The goal was to group individuals into categories; each category contains individuals who are similar to each other and different from individuals in other categories [6].

To understand which factors influenced health heterogeneity and the impact of health heterogeneity’s impact on healthcare utilization, two main research programs were conducted in Taiwan, and the previous research was reviewed. First, we examined the socio-demographic and economic characteristics associated with the health profiles of elderly people, and secondly we examined the effects of different health profiles on the utilization of and expenditures for healthcare services. We believe that the person-centered approach, which took unobserved heterogeneity into account [6], could be valuable in understanding the burden of heterogeneous elderly people (health profiles) on the health care system, and provide new perspectives for policy makers to develop measures for the evaluation of trends and outcomes in order to monitor the needs for care, and to improve quality of care and efficiency at the national level.

## 2. Universal National Health Insurance (NHI) In Taiwan

A universal national health insurance (NHI) program was initiated in Taiwan in 1995. By June 2011, more than 99% of the total population (approximately 23 million people) had benefited from NHI. According to the statistics [8], nearly 35% of the total health care expenditure of NHI in Taiwan was used by elderly people aged 65 and over. Previous research has shown that the three most frequent principal diagnoses for elderly ambulatory care visits were diseases of the circulatory system (17.3%), the respiratory system (15.9%), and the musculoskeletal system and connective tissue (12.8%) [9]. On average, the number of ambulatory care visits by elderly people in Taiwan was 26.8 per year, while the average was 15 visits among all age groups in 2011, which has recently raised concern by officials with the NHI [8]. Nearly all elderly people utilized ambulatory care services and half made more than 24 visits annually to ambulatory care centers; this is a much higher number of visits than reported in the United States and other Organization for Economic Cooperation and Development countries [9,10]. Previous research has shown that the availability of Taiwan’s NHI has led to greatly increased use of both outpatient and inpatient services by elderly people [11]. The statistics also showed that elderly persons were the main consumers of Taiwan’s NHI [12].

In addition, long-term care is presumably in increasing demand by elderly people, particularly those with functional decline. In Taiwan, long-term care is now covered by the policy of a “Ten Year Long Term Care Plan”. This welfare service was launched in 2007; by 2012, about 22% of elderly people requiring long-term care were receiving the service [13]. Since NHI covers all medical care costs with limited co-payments up to 10%, and has recently extensively reimbursed some intermediate-care and long-term care services, such as rehabilitation, home nursing care, and other services [8], it is possible that some long-term care needs of elders were partially shifted to and covered by NHI.

## 3. The Concept of Health Heterogeneity and Health Profile Identification

It has been well documented that the group of elderly people actually consumes a disproportionate—and sometimes inappropriate—amount of services [8,14]. Thus, when dealing with the healthcare needs of elderly persons, it has become increasingly important to consider their health status holistically, using an individual-based, person-centered approach. To describe health heterogeneity more comprehensively, this approach takes all possible observed health indicators into account. There is also growing recognition of the need to evaluate how healthcare services are delivered and received, and how the healthcare system might best be enhanced to meet the health needs of an aging population [15].

Using the 2005 National Health Interview Survey (NHIS) dataset of Taiwan, all available health indicators were used in our studies to determine health profiles of elderly persons by LCA. The health indicators chosen were related to need factors as measured by health conditions, sensory limitations, and functional impairments as the potential endogenous factors that influence the health of elderly people. Indicators (yes/no) of chronic conditions included self-reported hypertension, diabetes, renal disease, heart disease, stroke, cancer, respiratory disease, joint and musculoskeletal problems, and other comorbid conditions. We included only those conditions where individuals acknowledged that the diagnosis had been confirmed by a physician. Sensory limitations (yes/no) included self-declared problems with hearing and vision. Self-rated health (bad/not bad) was determined by the interviewee’s own perception. Cognitive problems were measured with the Mini Mental State Examination (MMSE; scores < 23 indicated cognitive impairment). Depression was examined with the Geriatric Depression Scale (GDS-15; scores > 10 indicated depression). Functional disability (yes/no) was defined as difficulty with activities of daily living (ADL), including eating, taking a bath/shower, dressing, using the toilet, getting up from a bed/chair, moving around the home; instrumental ADL (IADL), including cooking, shopping, using the phone, taking medication, doing light and heavy housework, washing, managing money and degree of mobility (difficulty in performing upper and lower limb movements).

**Figure 1:**
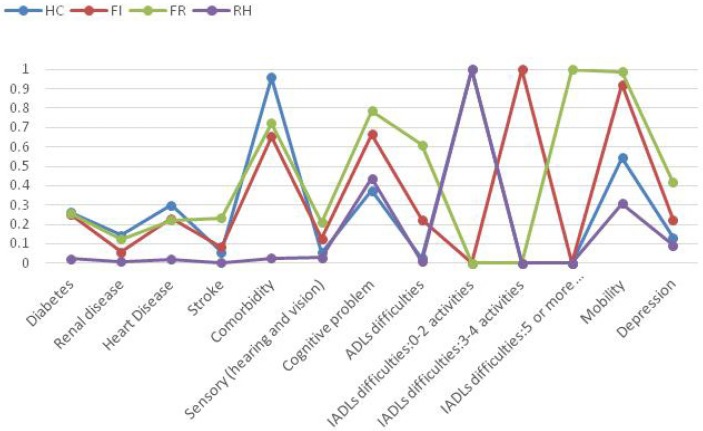
Predicted probability for health indicators, conditioned on latent profile [19].

A previous study that evaluated 1,064 older Canadians used LCA to identify four latent classes of health status, including an apparently healthy group, one with physical impairment, one with psychological impairment; and a group who had both physical and psychological impairment [14]. In Taiwan, four health profiles were also distinguished by using LCA (Figure 1). Within the context of Taiwan’s health care system, the first group was characterized by high probabilities of various diseases (diabetes, heart disease, and other comorbid conditions), but with relatively low probability of cognitive problems, little difficulty performing ADL, and no difficulty performing IADL. Because of their high comorbidity, we labeled this group as a “High Comorbidity” (HC) group. The second group was labeled as the “Functional Impairment” (FI) group. They had relatively high probabilities of having difficulty with ADL, high probabilities of having difficulties with mobility and with cognitive problems. In the third group, individuals had the highest probabilities of having physical (with high difficulties in ADL, IADL, and in mobility) and cognitive impairment, as well as depression. They also had various diseases and multiple chronic conditions, which made them the most vulnerable group. Because of this, we labeled this group as the “Frail” (FR) group. The last group of elderly individuals was labeled as the “Relatively Healthy” (RH) group. This group was comparatively healthy and less likely to report chronic diseases, comorbid conditions, cognitive disorders, or depression. They also had low probabilities of disability and relatively few functional limitations. Among the elderly people in Taiwan, nearly 43.5% fall into the HC group; about 6.2% fit into in the FI group, 10.3% are classified in the FR group; and 40 % in the RH group.

According to results of the 2005 National Health Interview Survey (NHIS), which was conducted for elderly people in Taiwan, nearly 17% of elderly people (the FR and FI groups) may need healthcare assistance due to functional disability. This percentage is higher than the 12.7% of those who had trouble with at least one ADL, according to the same survey. It is also higher than another population-based estimation in 2008, which showed that approximately 15.02% of older Taiwanese may need health care assistance. That survey used only three variables (ADL, IADL, and the Short Portable Mental Status Questionnaire (SPMSQ)) as health indicators [16]. Since the percentage of elderly people who were with at least one ADL difficulty has been moderately increasing over the past 10 years [17], it is evident that when taking health heterogeneity into account by incorporating more comprehensive health indictors, there may be actually more elderly people who need care.

Heterogeneity in elderly people is not a new concept. However, with complex chronic conditions, diseases, and functional disabilities, it is critical that all health complaints, both physical and psychological (such as depression, and worsening cognition), should be taken into consideration together if we are to meet the increasing healthcare demands of older people [18].

## 4. Socio-Demographic and Economic Determinants of Health Heterogeneity

Previous research showed that different socio-demographic and economic characteristics were risk factors influencing elderly peoples’ health status and health care utilization [20,21,22]. For example, research indicated that the health status of elderly people deteriorates with aging, and age was one of the most important factors in health care utilization [15,22,23]. Gender differences were also reported. Elderly females reported poor self-rated health, greater cognitive impairment, and poorer scores on ADL and IADL than did elderly males. Women also have twice as much comorbidities and symptoms of depression, and three times as many limitations in mobility [24]. Widowhood, together with economic difficulties, is associated with a higher risk of depression in men [25], and research also suggested the relationship between living arrangements and health for men and women [26]. Socioeconomic status (SES) was also found to be significantly associated with health status [20,22,27]. We believe that socio-demographic and economic characteristics related to social capital, defined as “the ability of actors to secure benefits by virtue of membership in social networks or other social structures” [28], also had a capital impact on the health inequality of elderly people. In terms of ethnicity, the authors of a previous study mentioned that among older Taiwanese, ethnicity (*i.e.*, Fukienese, Hakka, or Mainlander) was an important predictor of differences in subjective ranking of social status [29]. The inequality found among these groups correlates with health outcomes, including mortality, disability, and self-rated health [30,31].

In Taiwan, the sociodemographic differences were significant among elderly persons with the four health profiles. It was clear that, with increasing age, elderly people in the HC, FI, and FR groups were more likely to be vulnerable and to need care assistance. Apart from age, individuals in the HC, FI and FR groups, in comparison to the RH group, were more likely to be female. Those with advanced age and female gender had increased likelihood of being in the FR group and less likelihood of living alone or with a spouse only. Apart from the increasing likelihood of age and gender (female), the FI group was also less likely to live alone than were elderly persons in the RH group. Elderly persons in the HC group were more likely to live in urban areas, to be middle class (with a monthly income of NT$30,000 to NT$50,000) and to be male. When members of the HC group were compared further with the FI group, they tended to be younger and also more likely to have migrated from Mainland China. We also found that elderly Hakka were less likely to be categorized in the FI and FR groups, which indicated that they were more likely to have better health than do their Taiwanese counterparts. This may be partially related to the characteristics of Hakka, who are known to be hardworking and thrifty, and an area that needs to be further addressed in the future.

Previous research, both cross-sectional and in some longitudinal studies, has reported that older adults living alone are at greater risk for poor physical and emotional health [32] and cognitive decline [33]. However, other studies found that those living alone may actually have health advantages. For example, a study of health among the oldest-old in China showed that living alone and living with children are associated with both health advantages and disadvantages [34]. The literature is not clear whether older adults living alone have health disadvantages or whether co-residential arrangements are beneficial to their health. Some underlying factors may cause the inconsistency, such as different interpretation of living arrangements and the culture of study samples. However, after controlling for different health profiles using the person-centered approach, elderly people in the FR and FI group were less likely to be living alone. From the point of care, there is correlation between living arrangements and health profiles. In other words, lack of the ability to live alone may be the issue for frail elders and living arrangements become a protective factor. In Taiwan, those frail elders with functional impairments were less likely to be able to live alone without the help from a social network.

The health effects associated with different living arrangements may also vary by gender. For instance, it has been reported in western studies that men benefit more from marriage than do women [35]. Another study found that women living alone had a lower risk of decline in mental health and vitality compared with women living with a spouse [36]. Prior gender-stratified analyses have suggested a different pattern of associations between living arrangements and health for men and women [26,37,38]. Although we did not examine the effect of marriage, due to its high co-linearity with living arrangement, our finding was consistent with previous research that regardless of gender, elderly people in frail groups had a reduced likelihood of living alone and that frail men were significantly more likely to live within a family network. To identify circumstances influencing living arrangements and health from gender perspectives was critical to facilitate gender-sensitive services.

Educational levels were also important factors associated with the health groups [39,40]. Elderly people with more educations were less likely to be either in the FR or the FI group in Taiwan. Although economic status was not significantly correlated with the FR and FI groups, middle class individuals who lived in metropolitan/urban areas were more likely to be in the HC group; this was especially significant among elderly men. It may be that in Taiwan, living in urban areas with more healthcare resources (e.g., hospitals and medical clinics), compared with living in rural areas, made it easier to express health care needs. In general, previous research indicated the relationship of socioeconomic status to health [19]. Some also showed the relationship of income to health, after controlling for the factor of education [41,42,43]. Based on the concept of social capital defined by its function [44], social capital is productive and facilitates certain actions of individuals who are within the social structure. The socioeconomic status, living arrangements, and residence were related to the elder’s personal and social capital. We believe that social capital, defined as “the ability of actors to secure benefits by virtue of membership in social networks or other social structures” [28] and the “information, trust, and norms of reciprocity inhering in one’s social networks” [45] make it possible to achieve certain ends that would not be attainable in its absence [44]. 

The heterogeneity of elderly people shown by their health profiles reflects the differences and socioeconomic characteristics of the current elderly generation in Taiwan. The causal relationship of these characteristics was critically important both for identifying the high-risk groups of elderly people and for appropriate health care resource allocation.

## 5. The Effect of Health Profiles on Utilization of Health Care Services

In terms of utilization of health services, one of the most frequently used frameworks has been the behavioral model developed by Andersen, Aday, and others. This behavioral model, which has been extensively revised [46], points to multiple influences on the utilization of healthcare and patients’ health status. It uses a systems perspective to integrate the characteristics of an individual (including predisposing, enabling, and need factors), and contextual (environmental and provider-related) variables associated with decisions to seek care [47]. Based on the emphases of the health and socio-demographic and economic factors in the model [46], it is useful to identify the socio-demographic and economic characteristics associated with the health status of elderly people. In concept, the contextual variables would influence individuals’ use of health care and would show that outcome, in turn, affects predisposing, enabling, and perceived needs for health services as well as healthcare seeking behavior by way of feedback loops [46].

The heterogeneous group of elderly people actually consumes a disproportionate and sometimes inappropriate share of healthcare services [14]. Previous research also showed that factors of need, objective and perceived, were stronger than were other variables [46], an effect that persisted after controlling for potential endogenous factors [48]. The health profiles encompass multiple dimensions of health, and capture their likely synergistic effect on the overall health needs of older individuals [14]. For elderly people with complex care needs, comorbidity has now become the rule [49], and the extent to which each disease and syndrome translates into disability and service utilization varies greatly [14,50,51]. Lafortune *et al.* (2009) suggested that exploration of the heterogeneity of elderly people with a person-centered approach using homogenous health state categories combined with their socioeconomic characteristics provided a valid basis for comparing configurations of service utilization [14].

**Figure 2:**
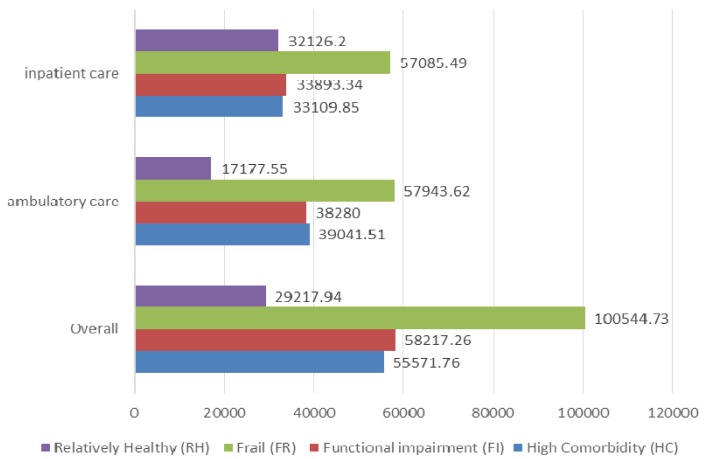
The annual mean health care expenditures for each health profile (per user/per year).

The effects of health heterogeneity on the utilization of healthcare services were explored in Taiwan [19]. The effect was significant for both the likelihood of utilization and its cost (Figure 2). The FR group was apparently included the heaviest users of health care, due to their combination of physical, cognitive, and functional impairments. Among the heavy users of ambulatory care services, the HC group had the highest likelihood of utilizing the ambulatory care services of the NHI. The FR group, the HC group, and the FI group also had higher likelihoods of being hospitalized and actually accounted for more expenditure for healthcare services when compared with the relatively healthy group.

Previous research has shown that the availability of Taiwan’s NHI greatly increased the utilization of both outpatient and inpatient services by elderly people [11]. The statistics also showed that elderly people were the main consumers of Taiwan’s NHI [12,52,53]. Since Taiwan’s NHI covers medical care needs in the acute care system, the “frail” elders were expected to be heavy users of care. However, the HC group was identified as just as likely to utilize the ambulatory care services of NHI as the other two groups, whereas the relatively healthy group did not do so significantly. NHI provides easy access for those in need, and this phenomenon could be partially explained by previous research that showed that those who had coverage for a particular service were more likely to use that service [54]. Since NHI covers all medical care costs with limited co-payments, and has recently extensively reimbursed some intermediate care and long-term care services such as rehabilitation, home nursing, and other services, the long-term care needs of elders were quite possibly partially shifted to and covered by NHI at this stage.

## 6. Healthcare Expenditures in Each Health Profile, by Age and Gender

Previous studies have shown that the older age population usually accounts for the biggest proportion of a country’s healthcare costs [55,56] and that age is one of the most important factors in healthcare utilization [15,23]. In our study, the significant differences in healthcare expenditures for each health profile were evident; however, age was no longer a prominent factor after controlling for the health profile. The effect of health profiles had the least impact on the oldest-old (aged 85 and over). Our study suggested that age was influential only for the likelihood of hospitalization and not for healthcare expenditures [19]. These findings were consistent with previous research in The Netherlands suggesting that age may not be the primary cause for healthcare expenditures, but instead may act as a proxy for the health status of elderly people [57]. Only for the oldest-old, the effect of health profiles became less significant. This may be explained in part by their being closer to the end of their life, and the effect of “time to death” instead of age determines expenditures on acute care [58,59,60].

In terms of gender, males were more likely to use inpatient care services and less likely to be heavy users of ambulatory care than were females. This may be related to different behavioral responses by males and females in their contextual environment [61]; however, for health care expenditures, these variables showed no further significance after controlling for health profiles. It may be that healthcare expenditures related to prescriptions are constrained more by clinical gatekeepers, such as physicians, and may partially account for the male-female health-mortality paradox, and is a topic that needs further study. This suggests that when policy makers deal with the issue of aging, it is meaningful to explore the heterogeneity of elderly people with a person-centered approach rather than to automatically assume that only age and gender are important.

## 7. Other Factors Associated With Health Care Expenditures in the Health Behavior Model

While health profiles have strong effects on the utilization of healthcare services and subsequent expenditures, other variables also have a significant influences but their magnitude is determined by the nature of the health measure employed [48]. Based on the Anderson health behavior model, other factors associated with expenditures for healthcare services found in Taiwan included higher socioeconomic status and the experience of utilization of long-term care services after controlling for health profiles. It was evident in previous research in Taiwan showing that socioeconomic status does influence the individual’s health behavior [62]. For instance, previous studies have shown a strongly positive effect of income on the use of home health care [63]. Lee indicated that elders with higher education used more health services [64]. Evidently, more highly educated people tended to spend more on ambulatory care services, although they used fewer inpatient care services in Taiwan. However, the fact that economic status was not significant in the rate and costs of ambulatory care and inpatient care services, that is, no economic barriers to the health care system were shown also appears to reflect the relative fairness of Taiwan’s NHI. We argue that examining the use of health care services while controlling for health profiles may better reflect the true health care needs of elderly people and may also provide an alternative perspective for exploring factors associated with their utilization of health care services.

The experience of LTC utilization, either as formal or informal services, was found to be a risk factor for higher health care utilization. In Taiwan, the experience of LTC utilization had a significant influence on ambulatory care expenditures and inpatient care use. These results were consistent with previous studies, in which a correlation was found between LTC use and hospitalization in the Netherlands [65]. One study in the U.S. also showed that a person who had a hospital stay within the previous year had more than twice the probability of using home health care compared to persons without a recent hospital stay [63].

According to Andersen *et al.* (1995) [46] the utilization of health care is also influenced by the characteristics of the health care delivery system. These include the volume and distribution of resources and organizational issues, including entry and structure. Currently, the main way of caring for elderly people in Taiwan is at home by their families [8] and long-term care expenditures were mainly paid for by elderly people themselves and their families, except for low-income elderly persons. To what extent elderly people would express their health care needs covered by NHI or other formal LTC services depends on the availability of informal care resources as well as on service delivery and financing [61].

## 8. Future Research

Although multiple health indicators serve as valid outcomes predictors, they relate differently to various dimensions of health. It becomes increasingly clear that health changes cannot be fully described by any one dimension [14]. As Crimmins indicated, not all dimensions of health have changed in the same direction at the same time, and trends in any one dimension are not evidence of health trends overall [66,67]. It will be important for future research to further explore the causal relationship of sociodemographic characteristics in order to identify high-risk elderly persons and for appropriate healthcare resource allocation. It is also critical that the male-female health-mortality paradox be further explored. In the long run, there is an increasing need to develop a systematic strategy to attack the issue of aging with a person-centered approach. In addition, providing care for the elderly should be addressed at the national level.

## 9. Conclusions

There is an increasing need to understand how aging affects health and vitality by means of a person-centered approach. That is, to describe health heterogeneity more comprehensively one needs to take observed health indicators into account. From this perspective, decision makers could assess the effects of heterogeneity and explore socio-demographic and economic characteristics, to better understand the health characteristics of elderly people, and thus to project their subsequent care needs and adjust the distribution of health care resources [14].

The increasing healthcare demands of older people and their subsequent utilization of health care services is a complex issue. It is evident that the effect of the different health profiles of elderly people on the likelihood of utilization and expenditure of health care services was significant. Taking the heterogeneity of elderly people into account can help provide a better understanding of the patterns of health care needs and health care use by the older population. By doing so, it will enable policy makers to develop a systematic strategy to attack the issue of cost of health care for aging populations. We suggest that a person-centered approach to aging is needed in order to satisfy current needs and to forecast expenditures in the future.
